# Long-term serial position effects in cue-based inference

**DOI:** 10.1371/journal.pone.0200821

**Published:** 2018-07-18

**Authors:** Ashley Lawrence, Rick Thomas, Michael Dougherty

**Affiliations:** 1 School of Psychology, Georgia Institute of Technology, Atlanta, Georgia, United States of America; 2 Department of Psychology, University of Maryland, College Park, Maryland, United States of America; University of Melbourne, AUSTRALIA

## Abstract

An important theoretical question in decision making concerns the nature of cue-generation: What mechanism drives the generation of cues used to make inferences? Most models of decision making assume that the properties of cues, often cue validity, initiate a set of dynamic pre-decision processes. In two studies, we test how memory accessibility affects cue use by manipulating both ecological cue validity and cue accessibility in a stock-forecasting task. Cue accessibility was manipulated by the pattern of accurate cue discriminations within experiment blocks of the learning phase of the experiments. Specifically, we manipulated the serial positions in which the cues accurately discriminated while holding overall cue validity constant. At test, participants preferred cues that discriminated early in the learning phase—a kind of primacy effect. The findings suggest that cue use is influenced by memory retrieval mechanisms and that cue use is not solely determined by cue validity. The results have implications for the development of computational models of heuristic decision-making.

## Introduction

Judgement and decision-making behavior is often characterized in terms of the use of heuristics, which are typically presented either as deviations from rational norms [1] or as ways to adapt to a decision environment [2]. Memory-based accounts of decision-making can explain a number of the standard heuristics and biases: availability, representiveness, base-rate neglect, overconfidence, and hindsight bias [3]. A natural extension of these memory-based accounts is to explicate the role of memory in heuristics, specifically, how cues are generated and utilized in the service of inference.

Most research within the area of adaptive decision heuristics and Multiple Cue Probability Learning (MCPL) have focused on cue-based inferences [4]. Cue based inferences are a type of decision in which a decision maker chooses the option they believe is higher on some criterion based on different attributes (cues) of the options. For example, a decision maker might try to determine which stock is more profitable based on attributes of the companies such as number of employees, location, and type of product. There are a number of models of how people make these types of decisions in terms of which cues they use and how the cues may be combined [5]. However, most models of cue-based inferences within the heuristics literature rely on the concept of cue validity [2]. Cue validity is typically defined as the proportion of times a cue correctly identifies the option higher on the criterion, given that it discriminates. A cue discriminates when one option has a different value for that cue than the other options.

Although most decision models within the heuristics literature make assumptions concerning the role of cue validity in dynamic pre-decisions processes, the most explicit model based on cue validity is the Take-the-Best heuristic (TTB). TTB assumes that people generate and search cues in the order of their validity and that people base their decision solely on the first cue that discriminates between the options. Although there is evidence that people can use TTB [6] [7] [8], there have been a number of criticisms, mainly relating to the assumption that cue generation and use is based on a pre-computed validity hierarchy [9] [10] [11]. Dougherty et al. [9] argued that cue validity is computationally complex and requires an automatic frequency counter assumption that is psychologically implausible. Moreover, Newell and his colleagues have argued that cue validity is difficult to learn via experience and that human decision behavior is often inconsistent with cue validity hierarchies [12][13].

To study the influence of accessibility on cue use, we developed a paradigm to test whether long-term serial-position effects (both long-term primacy and recency) occur within the context of a cue-based inference task. There is previous work that demonstrates how early experience influences later cue use. Perhaps one of the oldest learning effects is blocking, where an organism fails to learn the association between a neutral stimulus and unconditioned response when the neutral stimulus was always presented with a conditioned stimulus during learning. Blocking occurs because the new, to-be-learned cue did not add any new information to predict the onset of the unconditioned stimulus [14]. Similar to the blocking effect is the preexposure effect, whereby animals have difficulty learning a cue-outcome association if the cue was experienced in a prior non-predictive context [15]. More recent work within the MCPL tradition has documented a delayed exposure effect with probabilistic cues, where cues that are informative in early trials are learned better than cues whose validity manifest in later trials [16][17].

Although serial position effects have been demonstrated in probability judgment [18] and hypothesis generation [19] we believe our studies are amongst the first to establish such an influence on cue preference. This is theoretically consequential because it demonstrates the importance of memory and learning in cue-based inferences that is not currently accounted for in any modern cognitive-based theories of decision-making (e.g., the adaptive toolbox [5], HyGene [20], Parallel Constraint Satisfaction Model [21]). It is theoretically challenging to incorporate memory and learning processes into models of decision-making, which are typically centered on strategy selection. The lack of integration is surprising, however, given such memory and learning phenomena have been well-established for over 40 years.

Long-term serial position effects have been shown in list learning tasks in which words at the beginning and end of lists are more likely to be recalled than words in the middle [22]. In these studies, participants learn a number of word lists with a distractor task between the lists and are then asked to recall the words they learned after all lists have been presented. The typical recall in such a task is the expected long-term primacy, but counter to the modal model of memory, recency also obtains in such a task. Of course, the nature of the recency effect differs from standard recency, because it results from long-term memory; not from the maintenance of the last few items of a presented list in primary or working memory.

We extended this long-term recency and primacy paradigm to two experiments using a stock-forecasting task. For each experimental block, the serial position (early, middle, or late in the block) in which a given cue correctly discriminated was manipulated. Cues were paired such that cues within each pair had approximately the same validity but differed in the serial positions in which they correctly discriminated within each block. One member of the pair correctly discriminated between the dyads more frequently in the middle trials of each block and the other member of the pair correctly discriminated more frequently either at the beginning or the end trials of each block. By manipulating the position within each block that the cues discriminated while holding validity constant, we were able to directly test whether the distribution of correct discriminations affects cue use. Our primary dependent variable elicited the participants’ preference for cues explicitly, rather than indirectly inferring cue preferences from choice behavior. Specifically, participants had to select 1, 2, 3, or 4 cues to be used in the ensuing trial. This cue-preference measure seems a reasonable proxy to people’s subjective beliefs regarding cue usefulness. Although Rakow, Newell, Fayers and Hersby [23] used a similar measure in their Experiment 3, it is surprising that such measures have been rarely leveraged in decision-making research. The two experiments reported in this paper differed in terms of the validity of the two cue pairs, though conceptually the second study served as a replication of the first.

We predicted that participants’ cue use would be sensitive to overall cue validity as well as the position (early, middle, or late) in which the cues tended to discriminate across the entire set of lists. Moreover, we expected the effect of the distribution of correct cue discriminations to obtain even when the cues had the same validity. Specifically, we predicted that because of long-term recency and primacy, when the number of cues that participants could use was limited, they would select the cues that correctly discriminated more often in the early and late positions across the blocks. This prediction is based on memory encoding where the encoding of feedback associated with correct discriminations would be better encoded at either the beginning or the end of the block conferring an accessibility advantage to cues appearing in particular serial positions. Such a memory effect is similar to encoding mechanisms typically invoked to account for serial position effects in list recall and attentional shifts in reinforcement learning paradigms. Such results would provide evidence that cue accessibility influences cue use within decision heuristics.

## Experiment 1

The goal of this experiment was to determine whether the distribution of correct discriminations influenced explicit cue preferences in an inference-based decision task.

### Method

#### Participants

There were 22 participants in this study, recruited from the online experiment management system at the Georgia Institute of Technology. Participants received course credit for their participation and could receive $5.00 for correctly selecting the company with the highest stock price on one randomly selected trial from the test phase. The participants provided written informed consent to participate in this study, and the study was approved by the Georgia Tech institutional review board (IRB).

#### Procedure

Participants completed both a training phase and a test phase of a stock-forecasting task in which they were asked to predict which of two companies would have a higher stock price based on the attributes of the companies. The training phase consisted of 5 blocks of 15 dyad comparisons. The test phase consisted of 40 dyad comparisons. There were five dichotomous attributes (cues) that were associated with each company: asset rating (AR), earning potential (EP), liquidity appraisal (LA), optimized capital (OC), and profit intensification (PI). The validities of the cues were manipulated such that there were two pairs of cues with similar validities and one cue with a unique validity. The frequency with which each cue correctly discriminated in the beginning (first five trials 1–5), middle (trials 6–10), and end (last five trials 11–15) of each block of the training phase was manipulated as shown in Table 1. One member of each pair correctly discriminated more often in the middle of every block than either the beginning or the end (Cue 1 and Cue 3). The other member of the pair correctly discriminated more at the end (Cue 2) or the beginning (Cue 4) of each block. This was done to isolate effects of the serial position of cue discrimination on cue use while controlling cue validity within each pair. Note that Cue 5 is a filler cue that we did not specifically manipulate. To further explicate the method, notice that Cue 1 and Cue 2 have approximately equivalent validities, but Cue 2 has more correct discriminations in the end of each block compared to Cue 1. Cue 3 and Cue 4 both have equivalent validities, but Cue 4 has more correct discriminations at the beginning of each training block compared to Cue 3. If cue usage is dependent on serial order, then participants should choose Cue 2 over Cue 1 (recency) and should choose Cue 4 over Cue 3 (primacy). Overall discrimination rate (combining both correct and incorrect discriminations) was held constant for Cues1 through 4, meaning the values of individual cues differed between companies for about 60% of the trials.

**Table 1:** Frequency of correct discriminations by position within block for each cue.

	Cue 1	Cue 2	Cue 3	Cue 4	Cue 5
Validity	.91	.89	.71	.71	.58
Discrimination	.59	.59	.59	.59	.51
Beginning	14	8	10	20	6
Middle	16	6	13	6	10
End	10	25	8	5	6

**Fig 1:**
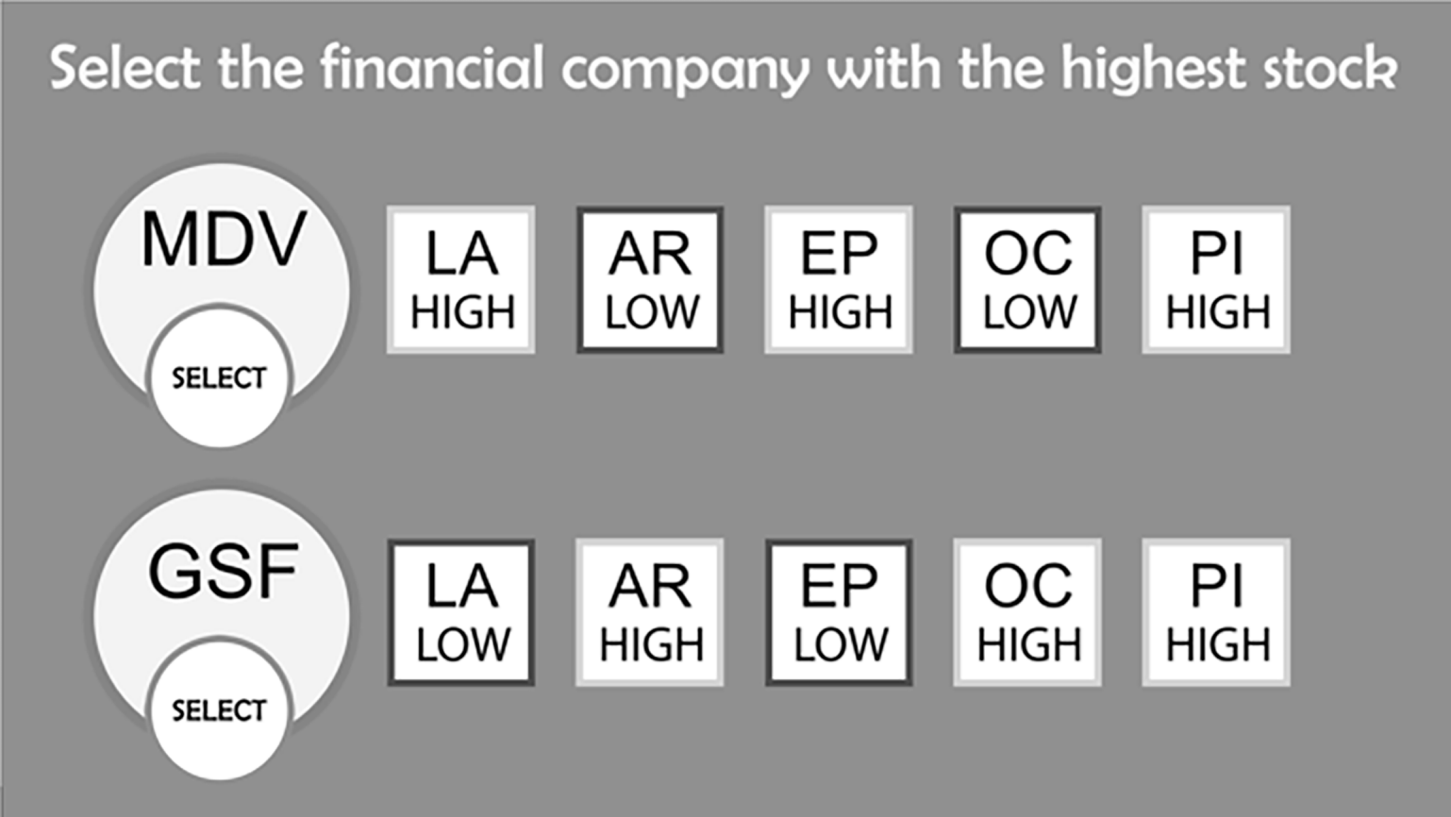
Example of a single learning trial in which all cues are visible.

During the training phase, participants were presented with 75 company dyads divided into 5 blocks. Each block was labeled as corresponding to a specific market sector: technology, financial, utility, property, or healthcare. There were no other differences between blocks than the label. The labels and the spatial locations of the cues were random between participants, meaning the cues listed in Table 1 did not appear in the same location or have the same label for all participants. The participants were asked to predict which of the two companies presented would have a higher stock value based on the attributes, as shown in Fig 1. Once they selected which company they thought would have a higher stock price they were given feedback on whether or not they were correct.

Between each of the blocks, participants completed a 25 second distractor task in which they were asked to verify simple mathematical equations involving addition or subtraction of single digit numbers. Each equation was presented for 5 seconds and the participants indicated whether or not the solution was correct.

**Fig 2:**
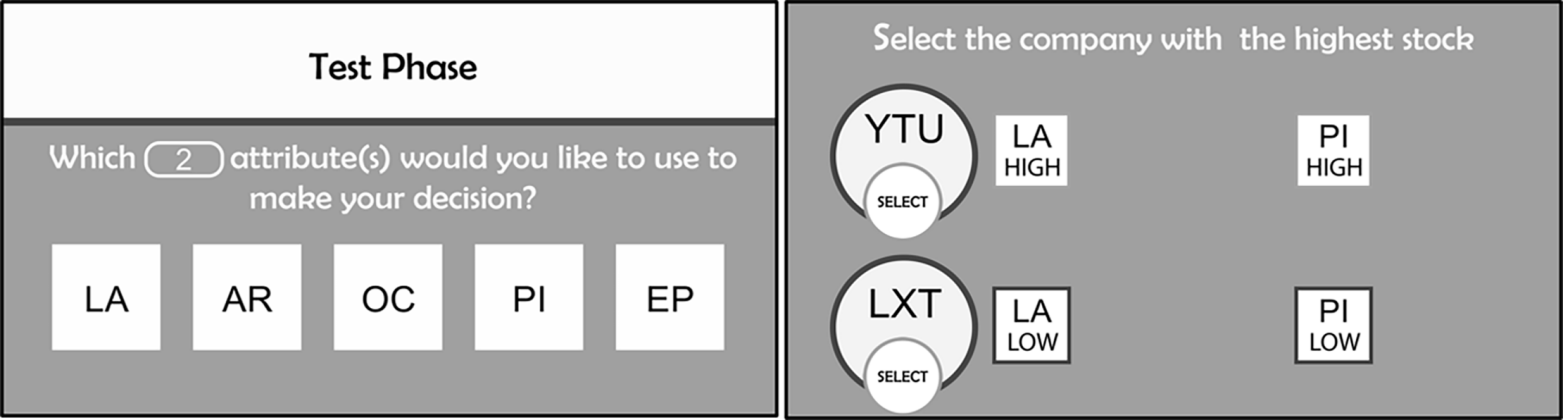
Example of a single test trial in which participants first select the cues they would like to use for their decision. Only those cues selected are visible during the decision.

In the test phase, participants were presented with 40 dyads and asked to indicate which of two companies they thought would have the higher stock value, similar to the training but without feedback. These companies were labeled as belonging to the service market sector, a sector not seen during training. Before comparing each company dyad, participants were told to select which cues they would like to use based on what they learned in the previous sectors. The number of cues that they had to select before they compared each dyad varied between 1 and 5. The number of cues participants could select was manipulated to determine participant cue preference. The participants were given 8 test trials (dyads) for each required number of cues. For the cases in which the number of cues was less than 5, the participants clicked on the cue labels to select the cues that would be revealed on the next screen during their choice between the dyads. Unlike in training, only the cues that the participant selected were visible as shown in Fig 2.

### Results

**Fig 3:**
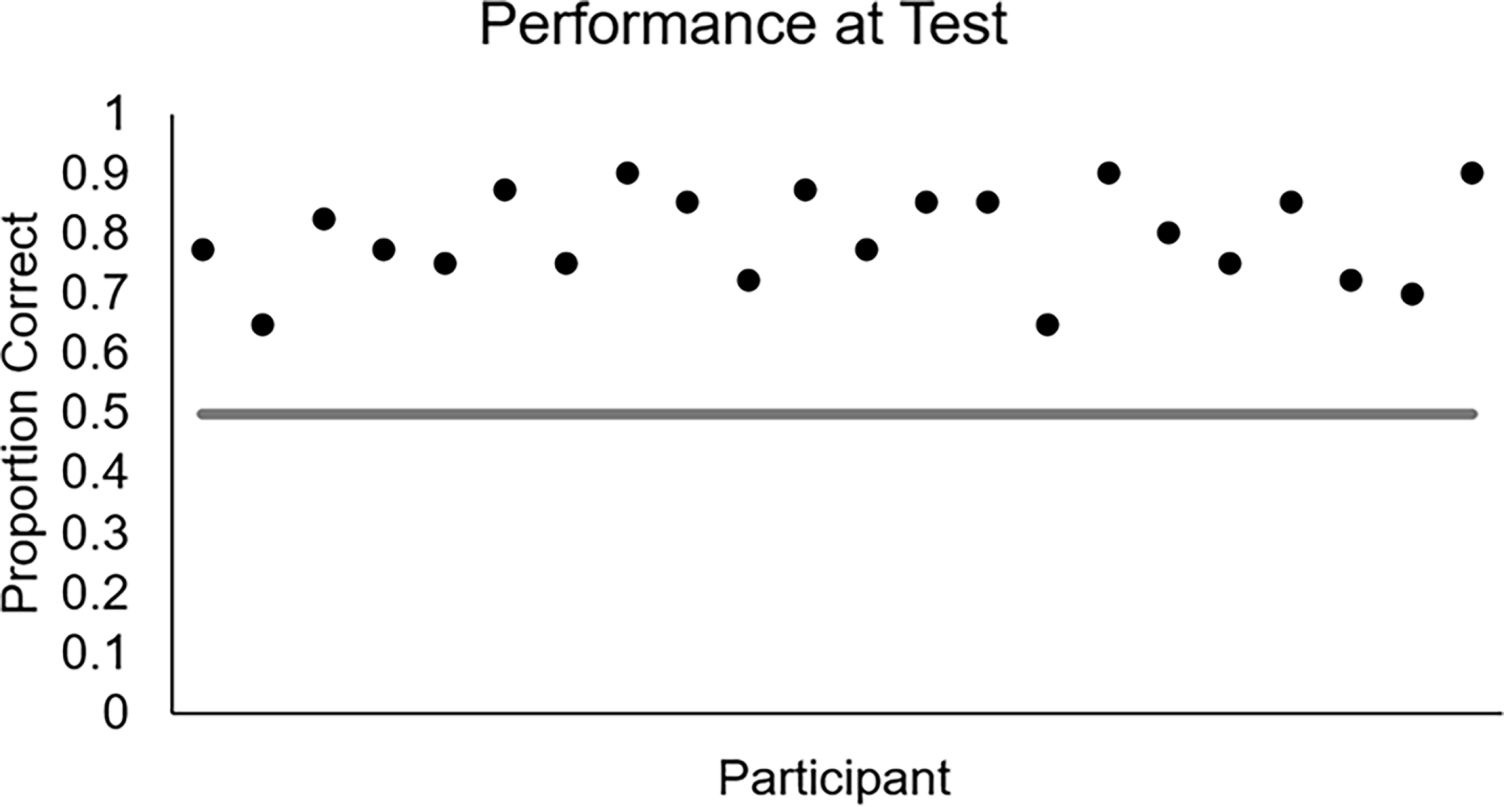
Proportion of correct responses during the test phase by participant.

In order to check whether participants learned during the task, a binomial test was conducted in which the proportion of correct responses during the test phase for each participant was compared to chance performance of .5. As shown in Fig 3, participants’ proportions of correct responses (.*80*) were significantly higher than chance performance, *z* = 17.53, *p* < .0001.

To further check learning, chi-square tests of goodness-of-fit were conducted to compare the proportion of trials in which each cue was selected. Specifically, in order to test whether participants were sensitive to cue validity, the proportion of selection for either of the two higher validity cues (Cue 1 or Cue 2) were compared to the proportion of selection for either of the two lower validity cues (Cue 3 or Cue 4), excluding cases in which none of the cues were selected or both a high and low validity cue were selected. This was done separately for all number of cues the participants were instructed to select and at the aggregate level. The selection of four cues was not included in this analysis because at that level participants always have to select one cue from each pair (proportions are always 1). For all numbers of selections less than four, the proportion of trials in which either higher validity cue was selected was significantly higher than the proportion of trials in which either lower validity cue was selected as shown in Table 2.

**Table 2:** Comparison of the proportion of trials in which either similar validity cue was selected by number of cues selected.

	Higher Validity (1 OR 2)	Lower Validity (3 OR 4)	χ^2^	P-value
N = 1	119 (80.95%)	28 (19.05%)	56.33	< .0001
N = 2	97 (80.83%)	23 (19.17%)	45.63	< .0001
N = 3	49 (76.56%)	15 (23.44%)	18.06	< .0001
N<4	265 (80.06%)	66 (19.94%)	119.64	< .0001

Note: Analyses not run on N = 4 because always selecting one from each set

Chi-square tests of goodness-of-fit were conducted to test the effects of the distribution of correct discriminations within blocks. Specifically, these tests were performed to determine whether the cues with equal validity were equally preferred. To satisfy the assumptions (i.e., mutual exclusivity) of the chi-square tests, the tests were conducted only for cases in which one or the other of each similar validity pair was selected. For example, tests of primacy were conducted for cases where either the middle cue (Cue 3) or the primacy cue (Cue 4) was selected and excluded cases where both or neither were selected. Similarly, tests of recency were conducted for cases where either the middle cue (Cue 1) or the recency cue (Cue 2) was selected. This was done to compare selection behavior to a null of .5 for both cues in each pair. The analyses were conducted separately by the number of cues the participants were instructed to select. To reiterate, cues within each pair had approximately the same validity but differed in the frequency with which the cues correctly discriminated by position within each block with Cue 1 and Cue 3 being more frequent in the middle, Cue 2 being more frequent in the end (recency), and Cue 4 being more frequent in the beginning (primacy).

**Fig 4:**
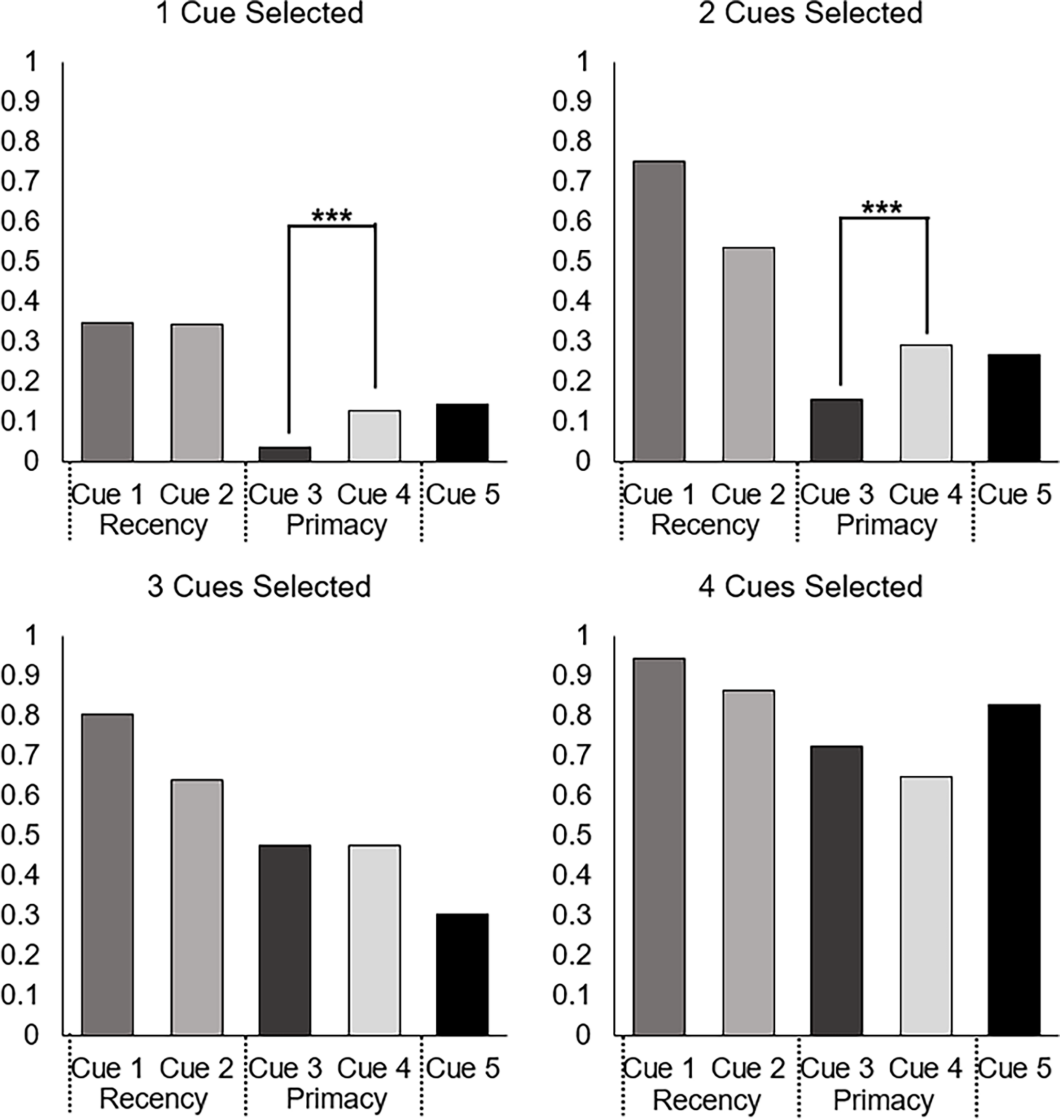
Proportion of trials in which each cue was selected by number of cues that could be selected. Evidence for a recency effect would manifest as a preference for Cue 2 over Cue 1, which was not observed. The preference of Cue 4 over Cue 3 is indicative of primacy. Note: *** indicates significance at *p* < .005.

As shown in Fig 4, the proportion of selection between Cue 3 and Cue 4 (χ^2^ = 9.14, *p* < .005) differed significantly from .5 when participants could only select one cue such that the primacy cue (Cue 4) was selected more often than its similar validity middle cue (Cue 3). When participants had to select 2 cues, the proportion of participants selecting Cue 3 and Cue 4 maintained the same pattern as what was observed for when participants selected a single cue (χ^2^ = 7.90, *p* < .005). When participants had to select 3 (χ^2^ = 0, *p* = 1) or 4 (χ^2^ = 1.55, *p* = .21) cues this pattern disappeared.

There were no significant differences in the hypothesized direction for cue selection proportions for the recency cue over the similar validity middle cue. In fact, there was a significant preference for the middle cue (Cue 1) over the recency cue (Cue 2) when participants selected two (χ^2^ = 17.51, *p* < .005), three (χ^2^ = 12.55, *p* < .005), or four (χ^2^ = 5.76, *p* = .02) cues.

### Discussion

Experiment 1 provides evidence for a memory-based account of cue selection behavior because there was a significant increase in preference for Cue 4 over Cue 3 when one or two cues were selected—a primacy effect. There was no indication of a long-term recency effect. It is also clear, that the participants were sensitive to the accuracy of cues. When comparing the two cue pairs directly, the more valid cues (Cues 1 & 2) were preferred over less valid cues (Cues 3 and 4). Moreover, participant performance during the test phase was above chance, providing further evidence that participants learned how to use the cues. The disappearance of the primacy effect when more than two cues were selected may have resulted from the general preference for the most valid cue pair such that once the two most valid cues were selected, participants did not show a strong preference among remaining cues. A second experiment was conducted to investigate this explanation.

## Experiment 2

Based on the results of the previous experiment, it seems possible that the difference in validity between the two cue pairs overshadowed serial position effects by creating a strong preference for the first two cues. In this experiment, the difference in validities between the cue pairs was smaller. We expected that the preference for more valid cues over less valid cues will be mitigated when the difference between Cues 1 and 2 versus Cues 3 and 4 is reduced. All other aspects of Experiment 2 were identical to Experiment 1.

### Method

#### Participants

There were 31 participants in this study, who were recruited using the same procedure as Experiment 1. The participants provided written informed consent to participate, and the study was approved by the Georgia Tech IRB.

#### Procedure

The procedure was the same as the previous experiment. However, the validity of the cues for this experiment differed from the previous one. As shown in Table 3, the cues had validities that were closer together than in Experiment 1. Moreover, the validities were manipulated so that the middle cue in each pair had slightly better cue validity to see if there was a serial position effect even when the ecology favored the middle cue. Again, the cues all had the same discrimination rate.

**Table 3:** Frequency of correct discriminations by position within block for each cue.

	Cue 1	Cue 2	Cue 3	Cue 4	Cue 5
Validity	.80	.77	.73	.70	.62
Discrimination	.59	.59	.59	.59	.56
Beginning	10	4	10	20	7
Middle	15	5	15	6	13
End	10	25	7	5	6

### Results

**Fig 5:**
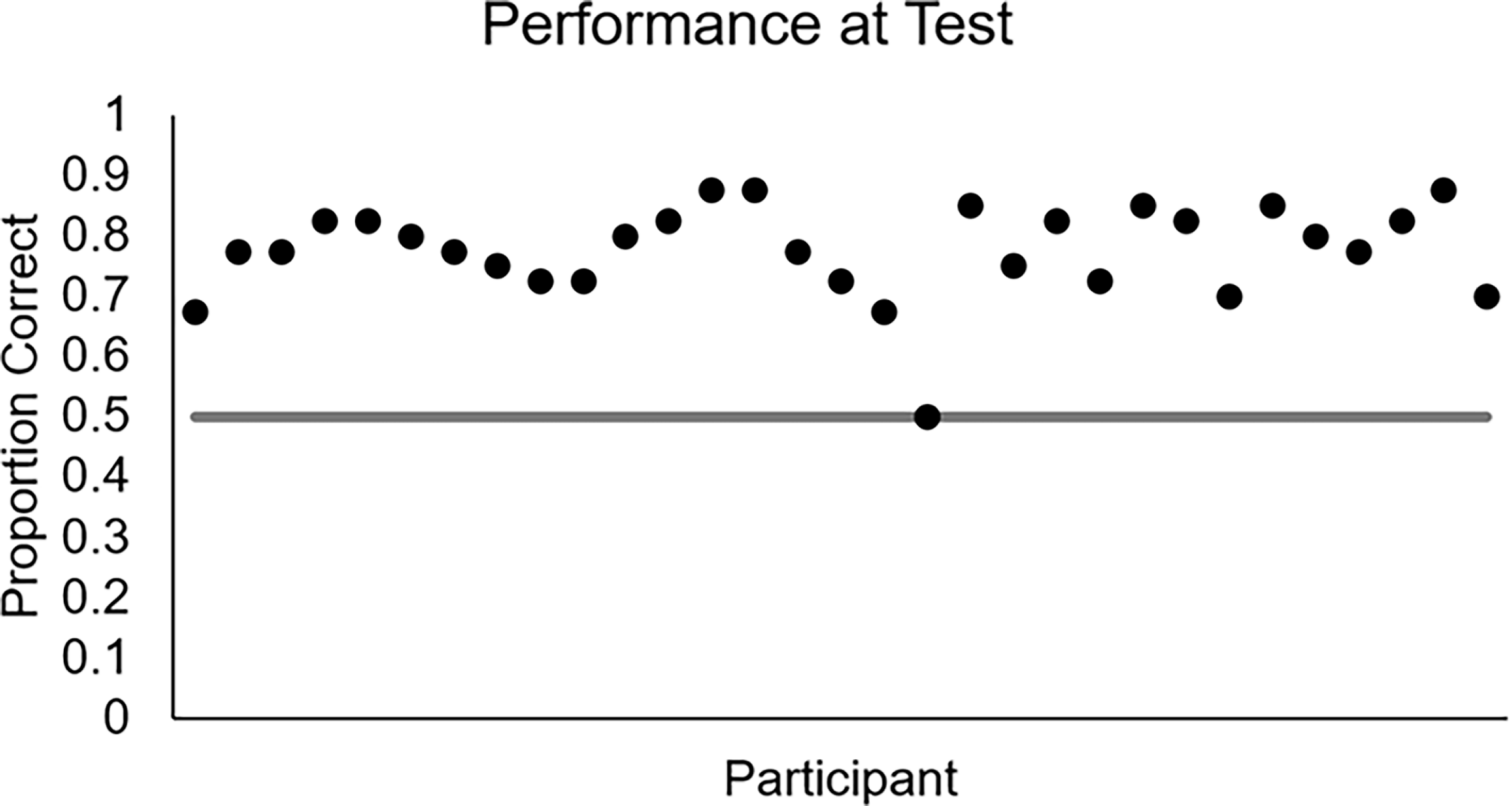
Proportion of correct responses during the test phase by participant.

A binomial test was run to evaluate participant performance during the test phase of this experiment. As shown in Fig 5, participants’ proportions of correct responses (.78) were significantly higher than chance performance, *z* = 19.42, *p* < .0001.

The same chi-square tests of goodness-of-fit tests used to further check learning in Experiment 1 were also conducted for this experiment. As shown in Table 4, the proportion of selection of either of the more valid cues (Cues 1 and 2) was significantly higher than the proportion of selection of either of the less valid cues (Cues 3 and 4) when participants were instructed to select one or two cues and at the aggregate level. However, the difference was not significant when participants selected three cues.

**Table 4:** Comparison of the proportion of trials in which either similar validity cue was selected by number of cues selected.

	Higher Validity (1 OR 2)	Lower Validity (3 OR 4)	χ^2^	P-value
N = 1	136 (67.33%)	66 (32.67%)	24.26	< .0001
N = 2	101 (66.01%)	52 (33.99%)	15.69	< .0001
N = 3	21 (52.50%)	19 (47.50%)	0.10	0.75
N<4	258 (65.32%)	137 (34.68%)	37.07	< .0001

Note: Analyses not run on N = 4 because always selecting one from each set

**Fig 6:**
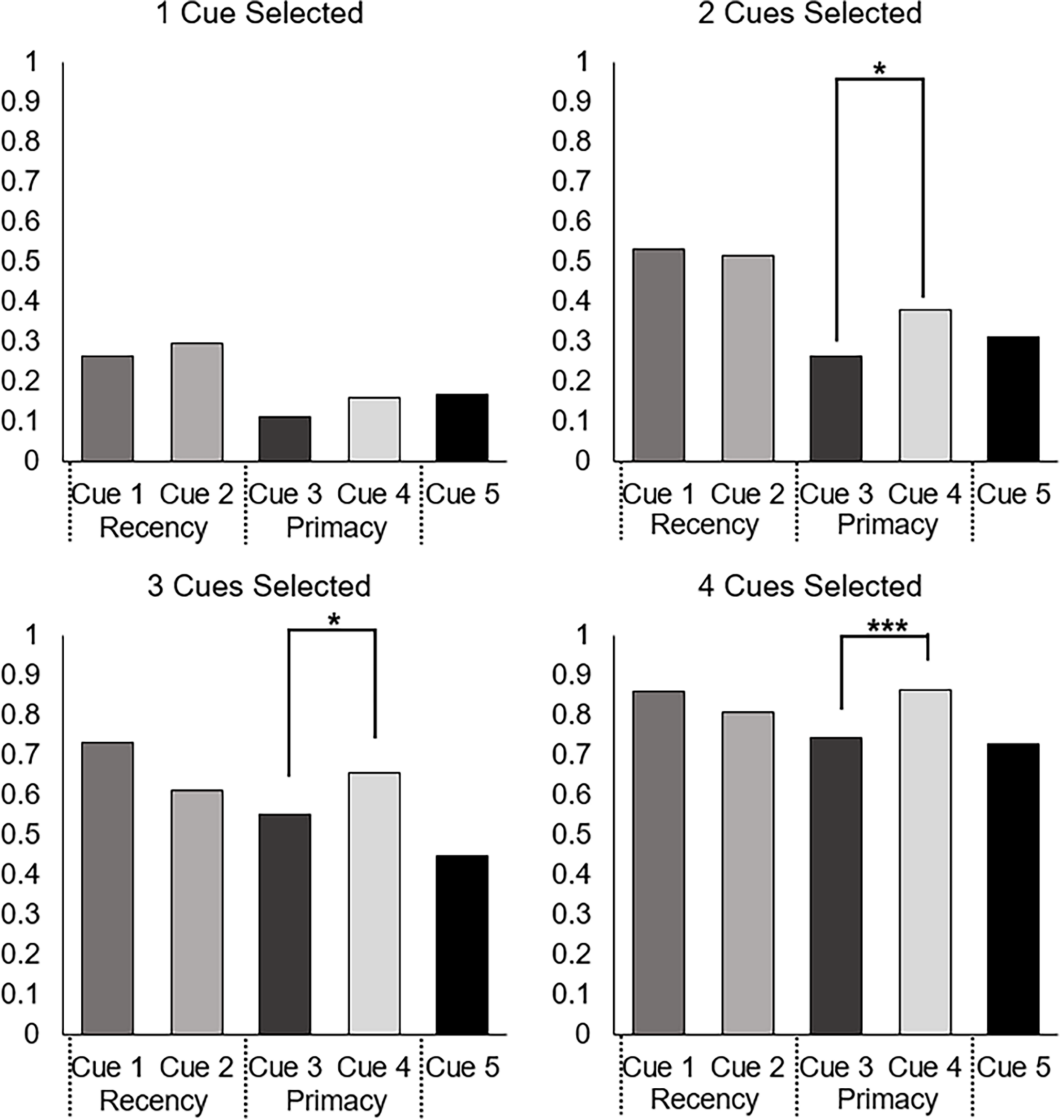
Proportion of trials in which each cue was selected by number of cues that could be selected for participant data. Evidence for a recency effect would manifest as a preference for Cue 2 over Cue 1, which was not observed. The preference of Cue 4 over Cue 3 is indicative of primacy. Note: *** indicates significance at *p* < .005, ** at *p* < .01, * at *p* < .05.

Similar to Experiment 1, chi-square tests of goodness-of-fit were performed to determine whether the cues with equal validity were equally preferred when only one member of the pair was selected. As shown in Fig 6, the proportion of selection between Cue 3 and Cue 4 differed significantly when participants selected two (χ^2^ = 6.03, *p* = .01), three (χ^2^ = 4.25, *p* = .04), or four (χ^2^ = 8.85, *p* < .005) cues such that the primacy cue (Cue 4) was selected more often than the middle cue (Cue 3). The difference was not significant for one cue selected (χ^2^ = 2.18, *p* = .14) despite the pattern being in the expected direction. Note, however, that the effect of primacy on cue selection for Cues 3 and 4 is opposite of what would be expected by a strict use of cue validity, as Cue 4 is actually slightly less valid than Cue 3. There were no significant differences in the hypothesized direction for cue selection proportions for the recency cue over the similar validity middle cue. There was no significant difference between cue selection when participants selected one cue (χ^2^ = .47, *p* = .49), two cues (χ^2^ = .12, *p* = .73), or four cues (χ^2^ = 2.09, *p* = .15). However, there was a significant preference for the middle cue (Cue 1) over the recency cue (Cue 2) when participants selected three cues (χ^2^ = 7.07, *p* < .01).

### Discussion

The results of this experiment, like Experiment 1, indicate an effect of long-term primacy on cue use but no effect of long-term recency on cue use. However, in this experiment, the pattern is consistent across all numbers of cues selected. Despite the middle cue having a slightly higher validity than the primacy cue, participants selected the primacy cue significantly more than the middle cue for all numbers of cues selected except one, providing additional evidence for an accessibility account of cue selection behavior. Yet, participants were still sensitive to the validity of cues such that they preferred the more valid cue pair over the less valid cue pair. Their above chance performance at test also indicates sensitivity to the accuracy of the cues.

## General discussion

The position of each cue’s correct discriminations within each block during training affected participants’ later cue use. Both experiments showed that participants were more likely to select the primacy cue, the cue that correctly discriminated more frequently in the beginning of each block, over the middle cue of similar validity. The serial position effect may be sensitive to the range of cue validities because primacy was more robust when the validities of all cues were more similar. Overall, we interpret the primacy effect as evidence for a memory-based account of cue use by extending long-term serial position effects to a cue-preference task.

It is important to note that neither experiment showed a long-term recency effect. This may be due to the relationship between long-term serial position effects and cue validity because the cue pair testing recency was always the most valid. However, it could also be the case that a type of blocking, preexposure, or delayed exposure effect is responsible for the lack of recency. Kruschke’s [24] models of attention shifts could explain the lack of recency where cues that discriminate early attract attention at the expense of attending to cues that discriminate later. If this is the case, however, then it would demonstrate that the delayed exposure effect can operate over shorter spans of trials than previously shown. Future research could address this issue by manipulating primacy and recency between subjects as well as within subjects, while holding cue validity constant.

Previous studies on the influence of memory within the process of cue generation [25] [26], have found that decision strategies were not consistent with TTB when the least valid cues were more salient or accessible. These studies focused on the effect of memory on strategy-use within the adaptive toolbox rather than examining the generation and use of specific cues. In the studies reported here, the lack of differences in cue selection when the difference in cue validity was large may have been due to decisions strategies used by the participants. It could be that participants had only been using one or two cues to make their decisions so, when the number of cues they could select increased, they were less selective of the additional cues. The goal of the studies reported here was to examine the influence of memory on cue use while unconfounding cue accessibility and cue validity; thus, further research is needed to study specific strategies used under these conditions.

An alternative account consistent with strategy use in our task might be that decision makers were more likely to learn the good cues quickly in the task and then deploy executive attention to those cues that they learned predicted best in the early positions of the blocks. Although this argument is similar to the attentional shifts in Kruschke’s [24] model, attentional shifts in Kruschke’s [24] model are not necessarily the result of explicit guidance of attention via strategy use. Thus, a strategy-use account is not an encoding phenomena per se as much as a result from the implementation of a learned decision strategy.

One might argue that the cue selection behavior of the participants in our experiments reflects internal cue validity and that our findings serve to validate the construct of internal or subjective cue validity. While the concept of internal cue validity may seem reasonable psychologically, arguably it is unfalsifiable: In order to test a model that replaces validity with perceived validity, one must have a way of objectively defining perceived validity. This strikes us as problematic, since any ordering of cues (irrespective of their objective cue validity) could reflect ones subjective or perceived validities of cues. To illustrate, the same mechanism (perceived validity) could in theory be applied post hoc even if we observed primacy but not recency, recency but not primacy, or neither in our experiments. In other words, regardless of how the experiments could have turned out, a theory based on the assumption that people order cues by perceived validity would be supported. We argue that it is more fruitful to focus on developing cognitive process accounts of cue preference, generation, and use that provide testable and falsifiable predictions.

In summary, the findings show that understanding memory and learning processes is crucial for understanding decision making. We are not arguing that long-term memory primacy effects in word-list recall is the same in decision-making. Yet it is likely the case that our primacy manipulation affected how the cues were encoded during training or how attention gets deployed as a strategy emerges, resulting in differences in cue accessibility. This is an important point because most experiments on cue use within the context of decision making, including our own, have likely confounded cue validity and cue accessibility. Highly valid cues are more strongly associated with positive reinforcement feedback, resulting in an accessibility advantage for highly valid cues compared to less valid cues. Our experiment relied on serial position effects to isolate cue accessibility from positive reinforcement (cue validity)—the feedback associated with correct discriminations were better encoded at either the beginning of the block conferring an accessibility advantage to cues appearing in particular serial positions. Thus, such memory effects will have to be accounted for within the adaptive toolbox and alternative architectures to provide a full account of how cues are generated, valued, and used to make decisions.

## Supporting information

S1 File Experiment 1 training data. (CSV)

S2 File Experiment 1 test data. (CSV)

S3 File Experiment 2 training data. (CSV)

S4 File Experiment 2 test data. (CSV)
